# Anchoring on Hyperglycemia and Sepsis in the Presence of an Unforeseen Thyroid Storm

**DOI:** 10.7759/cureus.46138

**Published:** 2023-09-28

**Authors:** Andrew M Joseph, Monica Karas, Victor H Camba, Brian M Martin, John Preece

**Affiliations:** 1 Osteopathic Medicine, Nova Southeastern University Dr. Kiran C. Patel College of Osteopathic Medicine, Davie, USA; 2 Department of Research, Alabama College of Osteopathic Medicine, Dothan, USA; 3 Graduate Medical Education, Magnolia Regional Health Center, Corinth, USA; 4 Internal Medicine, Magnolia Regional Health Center, Corinth, USA

**Keywords:** management, diabetic keto-acidosis, sepsis, thyrotoxicosis, thyroid-storm

## Abstract

Thyroid storm (TS) is a relatively rare but life-threatening complication of an overactive thyroid that can manifest in a myriad of ways due to its multisystem involvement. Due to its relatively high mortality rate, it is essential that TS is recognized and treated promptly. TS can occur due to trauma, drugs, and sepsis. Identifying TS as a diagnosis is challenging to pinpoint due to its similar presentation to more common pathologies like sepsis and diabetic ketoacidosis (DKA). Here, we present a case of a 31-year-old African-American woman with type 2 diabetes mellitus following sepsis secondary to *Escherichia coli* pyelonephritis and DKA. Despite standard sepsis treatment, which included appropriate intravenous fluids and antibiotics, the patient did not improve. Further workup, utilizing the Burch-Wartofsky score, helped identify TS as the underlying cause of the patient’s hospitalization, despite no history of underlying thyroid disease. The inclusion of thyroid pathology as part of the differential diagnosis and workup of a patient with a sepsis-like presentation to avoid anchoring bias warrants further investigation.

## Introduction

Thyrotoxicosis is the pathological state of the thyroid inappropriately releasing thyroid hormone into the bloodstream leading to a cascade of signs and symptoms that appear as acute hyperthyroidism [1]. Thyrotoxicosis is a relatively benign condition that can be managed with radioactive iodine, medications such as thioamides (carbimazole, methimazole, and propylthiouracil), and beta-blockers (propranolol, atenolol, and metoprolol), or thyroidectomy. However, it can lead to a thyroid storm (TS), which is a potentially fatal complication if not rapidly diagnosed and treated [1]. TS can present with symptoms that originate from a myriad of organ systems such as high fever, tachycardia, hypotension, arrhythmia, anxiety, delirium, nausea, vomiting, and diarrhea [2]. The estimated mortality of TS has been noted to range from 8% to 25% of patients; as such, an elevated index of suspicion, along with early diagnosis and treatment, is critical for survival [2].

Due to the TS’ impact on a multiorgan level, the associated pathophysiology is not very well understood, and there is only a limited amount of literature on the common etiologies [3]. One such proposed theory suggests that a trigger, such as sepsis, results in an overactive sympathetic nervous system. Subsequently, this induces an excessive catecholamine release that causes an overproduction of thyroid hormone. This uptick ultimately causes a heightened end-organ cellular response that manifests as symptoms present in TS [3]. Trauma, drugs, and sepsis have been reported to lead to this potentially life-threatening complication [3]. In a case series, other identified etiologies included chest infection, urinary tract infection, and meningitis [4]. Each patient had reported some level of thyroid pathology including thyroid swelling, bilateral pedal edema, and jaundice, while only one patient had a history of hyperthyroidism [4]. Another case discussed a patient with a past medical history of uncontrolled hyperthyroidism and congestive heart failure who was later found to have a toxic goiter. [5] This patient eventually suffered but recovered from a sepsis-induced TS [5]. Per one systematic review, published in 2019, only 26 cases of concurrent TS and diabetic ketoacidosis (DKA) were found globally to date with a mortality rate of 15% [6].

TS has been described as a severe sequela of thyrotoxicosis that requires a triggering event to occur [2]. Because of this, correctly identifying and diagnosing TS can be challenging [2]. Due to the significant overlap between the features of TS and other acute medical conditions, objective methods such as the Burch-Wartofsky scale are used to establish a diagnosis of TS [2]. The Burch-Wartofsky scale provides criteria for TS and scores the functions of the nervous, thermoregulatory, gastrointestinal, and cardiovascular systems, with higher points corresponding to worsening dysfunction [2]. A score of greater than or equal to 45 is highly sensitive for TS, while 25-44 points is categorized as an impending TS, and less than 25 points is unlikely to be a TS [1,2].

Treatment of TS is mainly targeted towards blocking thyroid hormone production and its action [1]. The management of TS is multimodal and includes beta-adrenergic blockers, iodide, corticosteroids, and antithyroid therapy [1]. Examples of antithyroid therapy include propylthiouracil and methimazole [1]. The decision to utilize one or the other depends on the patient’s comorbidities and thyrotoxicosis severity. For life-threatening thyrotoxicosis, such as TS, propylthiouracil is preferred due to its ability to decrease T3 levels by nearly 45% in the first 24 hours, as compared to only 10-15% by methimazole [1]. For thyrotoxicosis, methimazole is preferred due to its lower incidence of major adverse events, when compared to propylthiouracil [1].

Since TS is considered rare but fatal, the diagnosis of this life-threatening complication is paramount [2]. The overlapping features of TS with other acute medical conditions such as DKA or cardiogenic shock make the diagnosis of TS very challenging [2,6,7]. We present the case of a 31-year-old African-American female with a past medical history of type 2 diabetes mellitus (T2DM) who initially presented with urosepsis and DKA before triggering a TS.

## Case presentation

A 31-year-old African-American woman presented to the emergency department with nine out of 10 abdominal pain that radiated to her right flank, which was associated with nausea, vomiting, dysuria, fever, and chills. She had a past medical history of T2DM and obesity, with a BMI of 32 kg/m^2^ squared (the normal range is 18.5-24.9 kg/m^2^), but had no chronic complications or any previous thyroid disease. Her family history included T2DM but no thyroid pathology. Her home insulin regimen included Insulin Lispro 10 units subcutaneously three times a day and Insulin Detemir 30 units subcutaneously at bedtime. She stated that she always took her insulin appropriately and checked her glucose three times a day. Physical examination of the patient was significant for a patient who appeared ill and in mild distress, with costovertebral angle tenderness over the right side of her back. Point-of-care glucose on arrival was 503 milligrams per deciliter (mg/dL) raising suspicion for DKA. Vital signs, including temperature, heart rate, respiratory rate, and blood pressure were measured, and are shown in Table 1.

**Table 1:** Vital signs of the patient when examined in the emergency room. Abnormal values are in bold. mmHg: millimeters of mercury; °F: degrees Fahrenheit; °C: degrees Celsius.

	Patient Value	Reference Range
Temperature	102.3 °F (39.06 °C)	97.8°F – 99.1°F (36.5°C – 37.3°C)
Heart Rate	160 beats/minute	60 – 100 beats/minute
Respiratory Rate	18 breaths/minute	12 – 18 breaths/minute
Blood Pressure	158/110 mmHg	90/60 – 120/80 mmHg

Initial labs, including the complete blood count and complete metabolic panel, were significant for an elevated white blood cell count of 15.3 x10^3^ per microliter (µL); normal is 4.5-11 x10^3^ per µL. Due to the patient meeting Systemic Inflammatory Response Syndrome (SIRS) criteria, a venous blood gas was ordered and is shown in Table 2. Based on the patient’s venous blood gas analysis, the patient was experiencing respiratory alkalosis.

**Table 2:** Initial venous blood gas of patient when examined in the emergency room. Abnormal values are bolded. mmHg: millimeters of mercury; mmol/L: millimoles per liter

	Patient Value	Reference Range
pH	7.477	7.31 – 7.41
PCO2	36.9 mmHg	41 – 51 mmHg
Bicarbonate	27.3 mmol/L	23 – 29 mmol/L

A urinalysis revealed urine glucose greater than or equal to 500 mg/dL (normal is 1-15 milligrams/deciliter (mg/dL)), ketones 20 mg/dL (normal range is < 20 mg/dL), positive nitrates, large leukocyte esterase, white blood cells too numerous to count, many white blood cell clumps, and moderate bacteria. A urine and blood culture were subsequently ordered. The toxicology screen was negative, plasma lactate level was 2.2 millimoles per liter (mmol/L) (normal range is 0.5-1 mmol/L) and lipase level was 16 international units per liter (IU/L) (normal is 10-140 IU/L). ECGs showed sinus tachycardia without ST segment changes.

**Figure 1:**
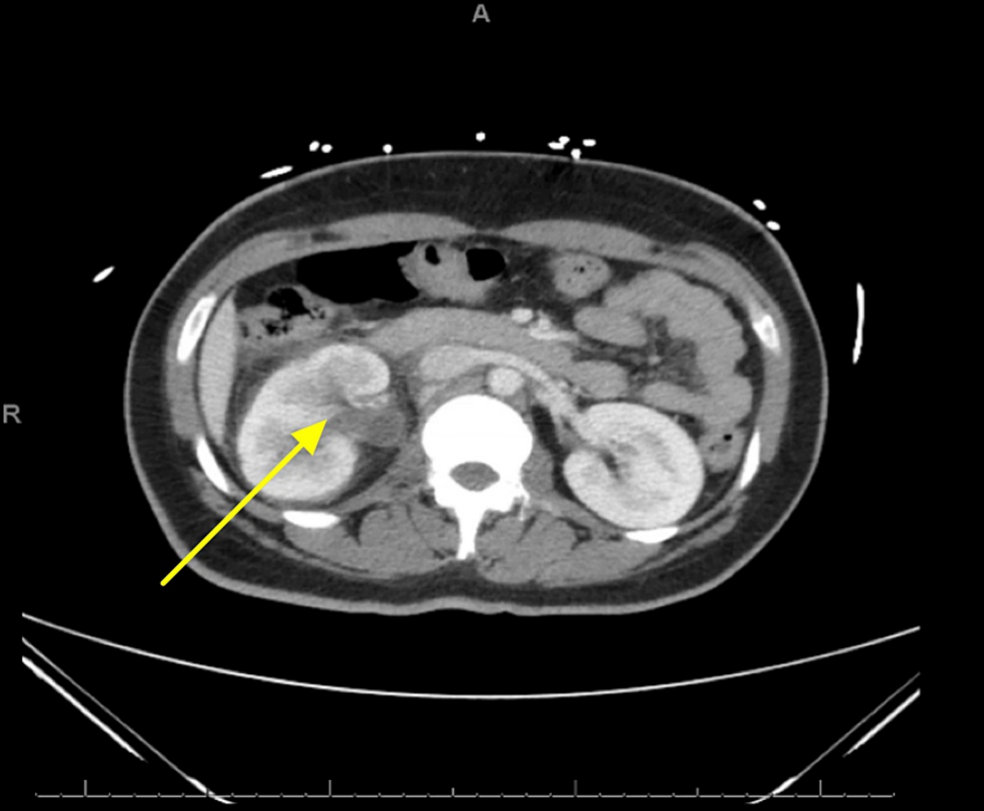
Computed tomography of the abdomen with contrast indicating mild right hydroureteronephrosis with suspicion of pyelitis or pyelonephritis.

A computerized tomography (CT) of the abdomen and pelvis revealed mild right hydroureteronephrosis, without obstructing stone. Additional findings were suspicious for right-sided pyelonephritis and pyelitis (Figure 1).

Additionally, the patient had mild DKA with concomitant respiratory and metabolic alkalosis suspected to be due to tachypnea and emesis, respectively. She also met sepsis based on the Systemic Inflammatory Response Syndrome (SIRS) criteria and pyelonephritis as the source and was treated with ceftriaxone and 1 L of IV lactated ringer. Additionally, her home insulin regimen of Insulin Lispro 10 units subcutaneously three times a day and Insulin Detemir 30 units subcutaneously at bedtime was restarted.

The patient continued to be febrile, with a peak temperature of 104.0 degrees Fahrenheit (40 degrees Celsius), tachycardic, with a heart rate ranging in the 130s-160s beats per minute, and tachypneic with a respiratory rate in the mid-20s breaths per minute. Additionally, her oxygen saturation had decreased to the high 80s-low 90s percent. The patient received an additional 3 L of IV fluids. Due to the persistence of her signs and symptoms, other causes were included in the differential diagnosis. Despite the patient not complaining of dysphagia or having pain in her neck, acute thyroid pathology was considered. Her neck was supple without tenderness to palpation or masses appreciated. No bruit was auscultated over her thyroid, and no hyperreflexia was noted. A thyroid-stimulating hormone (TSH) and free thyroxine (T4) level were ordered and revealed a TSH of 0.27 micro-international units per milliliter (µIU/mL) (normal range is 0.4-4.0 µIU/mL) and a free T4 of 2.21 ng/dL (normal range is 0.8-1.8 ng/dL). A thyroid ultrasound was performed which revealed a right thyroid lobe measuring 5.3 x 1.3 x 1.6 cm with no nodules or cysts, a left thyroid lobe measuring 5.5 x 1.4 x 1.9 cm with no nodules or cysts, and a thyroid isthmus measuring 5 mm. Overall, the thyroid was found to be of normal size (the normal range is 4-4.8 x 1-1.8 x 0.8-1.6 cm). Both the glandular vascularity and echotexture were found to be normal. A Burch-Wartofsky Point Scale was performed, and the patient fell in the “highly suggestive of thyroid storm” category of greater than 45 points (Table 3). The patient was immediately started on oral propylthiouracil 200 mg every 8 hours and potassium iodide.

**Table 3:** The Burch-Wartofsky score for clinically diagnosing thyroid storm in this patient. Patient's values are in bold.

Burch-Wartofsky Scoring Table	Point System	Patient’s Presentation
Temperature Fahrenheit (Celsius)		
< 99.0 (< 37.2)	0	102.3 F 20 points
99.0-99.9 (37.2-37.7)	5	
100-100.9 (37.8-38.2)	10	
101-101.9 (38.3-38.8)	15	
102-102.9 (38.9-39.2)	20	
103-103.9 (39.3-39.9)	25	
Greater than or equal to 104.0 (40.0)	30	
Central Nervous System Effects		
Absent	0	Absent 0 points
Mild (agitation)	10	
Moderate (delirium, psychosis, lethargy)	20	
Severe (seizure, coma)	30	
Gastrointestinal-Hepatic Dysfunction		
Absent	0	Moderate 10 points
Moderate (diarrhea, nausea/vomiting, abdominal pain)	10	
Severe (unexplained jaundice)	20	
Heart Rate (beats/minute)		
< 90	0	160 beats/minute 25 points
90-109	5	
110-119	10	
120-129	15	
130-139	20	
Greater than or equal to 140	25	
Congestive Heart Failure		
Absent	0	Absent 0 points
Mild (pedal edema)	5	
Moderate (bibasilar rales)	10	
Severe (pulmonary edema)	15	
Atrial Fibrillation Presence?		
No	0	No 0 points
Yes	10	
Precipitating Event?		
No	0	Yes 10 points
Yes	10	
Score Interpretation		
Unlikely to represent a thyroid storm	Score < 25	65 points highly suggestive of thyroid storm
Suggestive of impending thyroid storm	Score 25-44	
High suggestive of thyroid storm	Score > 44	

Despite IV fluids, the patient became hypotensive with her blood pressure ranging from the 60s-90s/40s-60s mmHg despite IV fluids. Therefore, the patient was started on a norepinephrine drip at 0.03 µg/kg/minute. The patient also received a hydrocortisone sodium succinate 100 mg every 8 hours.

The patient’s urine and blood cultures were positive for *Escherichia coli* which was sensitive to ceftriaxone. As such, the antibiotic was continued. Despite remaining afebrile, the patient continued to be tachycardic with heart rates between 90 and 120 beats per minute and had mild suprapubic abdominal pain.

**Figure 2:**
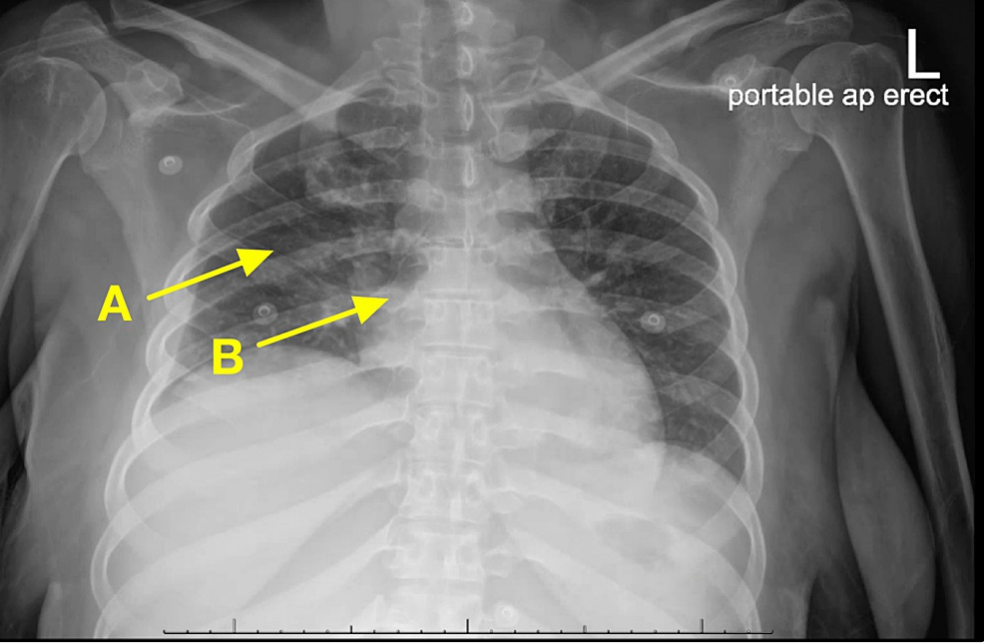
Chest X-ray revealing (A) hypoventilation with (B) resultant crowding of the pulmonary vasculature.

The patient became acutely hypoxic and was subsequently placed on high-velocity nasal insufflation with 30 L of oxygen per minute with a fraction of inspired oxygen (FiO_2_) of 50%. An arterial blood gas was performed which revealed a pH of 7.38 (normal is 7.35-7.45), partial carbon dioxide of 37.7 mmHg (normal is 35-45 mmHg), partial oxygen of 50.0 mmHg (75-100 mmHg), and bicarbonate of 22.6 mmol/L (normal is 22-26 mmol/L). A follow-up chest X-ray revealed hypoventilation with resultant crowding of the pulmonary vasculature (see Figure 2). A subsequent computed tomography angiography (CTA) of the chest revealed findings suspicious for multifocal pneumonia but with no signs of pulmonary embolism. A repeat electrocardiogram (EKG) showed no acute changes. Azithromycin was added to her medication regimen for additional pneumonia coverage.

Serum thyroid-stimulating immunoglobulins were less than 89% (normal range is < 130%); serum antithyroglobulin antibody concentration was less than 1 IU/mL (normal range is < 116 IU/mL); serum antithyroid peroxidase antibody concentration was 4.0 IU/mL (normal range is < 30 IU/mL).

Finally, the patient was weaned off the high-velocity nasal insufflation and eventually to room air. The patient was discharged home on methimazole 5 mg three times a day until she followed up with the endocrinologist, and cefdinir 300 mg twice a day to complete antibiotic therapy.

## Discussion

Initially, it was thought that this patient was experiencing DKA secondary to urosepsis. The patient was thought to have mixed respiratory and metabolic alkalosis secondary to tachypnea and hyperemesis, respectively, which masked the usual acidosis seen in this syndrome. The patient was treated with sensitivity-directed antibiotics, her home insulin regimen of Insulin Lispro 10 units subcutaneously three times a day and Insulin Detemir 30 units subcutaneously at bedtime, and IV fluids. Despite this, she did not improve and continued to be febrile and hypotensive. As such, thyroid function was assessed revealing a low TSH and elevated free T4 indicating acute primary hyperthyroidism, and TS was confirmed with greater than 45 points on the Burch-Wartofsky point scale. Propylthiouracil and potassium iodide were promptly administered, and the patient improved significantly.

The initial presentations of both TS and DKA appear eerily similar and include tachycardia, nausea, vomiting, tachypnea, abdominal pain, and headache [1,3]. Because of their similarity in presentation, there is the potential to incorrectly anchor on one diagnosis. Due to both potentially being fatal for a patient, it is critical for the care team to quickly identify the presence of either TS or DKA and begin management appropriately. Furthermore, identifying the root cause of either is equally as critical. One such cause of both TS and DKA is sepsis, which is an overactive and extreme response to an infection [8]. The presentation of sepsis is nearly identical to that of both TS and DKA, including tachycardia, tachypnea, and hyper- or hypothermia [9]. Because sepsis has the potential to be a life-threatening emergency, a set of criteria was established to rapidly make the diagnosis and start appropriate therapy [9]. Diagnosis includes the presence of an infection source and at least two of the following: a temperature greater than 100.4 degrees Fahrenheit (38 degrees Celsius) or less than 96.8 degrees Fahrenheit (36 degrees Celsius), heart rate greater than 90 beats per minute, a respiratory rate greater than 20 breaths per minute or an arterial carbon dioxide partial pressure (PaCO_2_) of less than 32 mmHg, or a white blood cell count greater than 12,000 per mm^3^ or less than 4000/mm^3^ or a band count of greater than 10% [8,9].

Since this patient met sepsis criteria, she was treated with appropriate antibiotics. However, the patient’s symptoms continued to persist, which indicated that DKA was probably not the culprit. Therefore, one of the main differential diagnoses was thyroid etiology. A low TSH and high T4 indicated acute primary hyperthyroidism, which was then confirmed with the Burch-Wartofsky point scale. Although there were no indications of a history of thyroid disorder, and the only medication the patient was on was insulin with which she was non-compliant, it can be concluded that the patient’s acute DKA episode and urosepsis triggered the TS, and not her medication. In literature, precipitating factors that are known to trigger TSs include abruptly stopping antithyroid therapy, thyroid imbalance prior to elective surgery, and labor and delivery [1]. However, these factors do not apply to this patient whatsoever. The patient was not on antithyroid therapies to begin with because she never had issues with her thyroid, the patient was not undergoing any elective surgeries, and she was not pregnant or in labor, or about to deliver. Therefore, having DKA and urosepsis as the etiologies of this patient’s TS are rare and unreported etiologies in the literature.

TS is a rare but known complication of acute and subacute thyroiditis [10]. There are three phases of thyroiditis, beginning with a thyrotoxic phase, then transitioning to a hypothyroid state before settling into a euthyroid phase [10]. In the thyrotoxic phase, the inflamed thyroid follicles release excess thyroid hormones into the circulation [10]. The excessive release of thyroid hormones can then lead to a TS [10]. This patient’s thyrotoxic phase of painless thyroiditis then led to the development of TS, but the main trigger event that started the cascade of painless thyroiditis leading to TS was DKA and urosepsis.

In this thyrotoxic patient with a nonnodular thyroid and no orbitopathy, other studies that would have been warranted in addition to the TSH and T4 levels include 1) thyrotropin receptor antibodies (TRAb) levels, 2) measurement of radioactive iodine uptake (RAIU), or 3) using a Doppler ultrasonography to determine the blood flow through the thyroid gland [1]. If the physical exam suggested a toxic adenoma or toxic multinodular goiter, then a thyroid scan and a 99mTc pertechnetate scan would have been indicated [1]. These tests can help distinguish Graves’ disease from other etiologies such as TS [1].

Since TRAb is cost-effective, administering it as an initial diagnostic test would have confirmed the most common cause of thyrotoxicosis, Graves’ disease, if positive [1]. In Graves’ disease, TRAb act as decoy TSH and stimulate the thyroid follicles to produce thyroid hormones [1]. The increasing levels of T4 in the blood result in negative feedback to the pituitary gland, which responds by the decreasing production of TSH in the blood [1]. However, the reduced TSH levels do not reduce the production of thyroid hormones because the TRAb molecules produced by Graves’ disease are stimulating the thyroid gland, acting as TSH [1]. Therefore, positive TRAb levels in the blood would strongly indicate Graves’ disease [1]. Yet if the TRAb levels are negative, then this only serves to rule out Graves’ disease, but not rule in any other etiology. Therefore, an RAIU would have been indicated if TRAb was negative [1].

RAIU measures the uptake of administered radioactive iodine over the course of 24 hours [1]. Diffuse elevated uptake is found in Graves’ disease, while it is usually normal but sometimes high in toxic nodular goiter [1]. RAIU will have no uptake in patients with painless, postpartum, or subacute thyroiditis [1]. Since this patient had painless thyroiditis, we would have expected the RAIU to have no measured uptake if it had been administered [1]. However, this patient was relatively contraindicated from undergoing RAIU because her poorly controlled diabetes renders her medically unstable for it [1]. Other conditions that need to be medically stabilized prior to starting RAIU include cardiovascular conditions such as atrial fibrillation or heart failure, pulmonary hypertension, infection, renal failure, pulmonary or cerebrovascular disease, and trauma [1].

## Conclusions

Due to the patient meeting sepsis and DKA criteria on the initial workup, there was an anchoring bias that resulted in providing management which did not yield substantial patient benefit. The resulting TS that transpired was then identified after reevaluating the patient and broadening the differential diagnosis. While the Burch-Wartofsky point scale is widely accepted as one of the leading tools to diagnose TS, its value is fully realized only when promptly utilized to enable immediate treatment implementation. Ultimately, the inclusion of a thyroid workup, including TSH, free T4, and the Burch-Wartofsky point scale, for a patient who presents sepsis-like symptoms warrants further investigation.
